# Comparative Study of Water Flow in Nanopores with Different Quartz $\left(10\bar{1}0\right)$ Surfaces via Molecular Dynamics Simulations

**DOI:** 10.3390/nano15120896

**Published:** 2025-06-10

**Authors:** Peng Zhou, Junyao Bao, Shiyuan Zhan, Xingjian Wang, Shaopeng Li, Baofeng Lan, Zhanbo Liu

**Affiliations:** 1 State Key Laboratory of Oil and Gas Reservoir Geology and Exploitation, Chengdu University of Technology, Chengdu 610059, China; zhoup@stu.cdut.edu.cn (P.Z.); baojunyao@stu.cdut.edu.cn (J.B.); wangxj@cdut.edu.cn (X.W.); liuzhanbo@stu.cdut.edu.cn (Z.L.); 2 Guizhou Energy Industry Research Institute Co., Guiyang 550025, China; lanbaofeng@gzmksj.com; 3 College of Energy, Chengdu University of Technology, Chengdu 610059, China; 4 School of Geophysics, Chengdu University of Technology, Chengdu 610059, China

**Keywords:** molecular dynamics simulation, quartz surface, water flow, nanopore, analytical model

## Abstract

Dewatering and gas production are applied on a large scale in shale gas development. The fundamental mechanisms of water flow in shale nanoporous media are essential for the development of shale oil and gas resources. In this work, we use molecular dynamic simulations to investigate water flow in two different quartz surface ($(10\bar{1}0)\text{-}\alpha$ and $(10\bar{1}0)\text{-}\beta$) nanopores. Results show that the $(10\bar{1}0)\text{-}\beta$ surface exhibits stronger water molecule structuring with a structure arranged in two layers and higher first-layer adsorption density (2.44 g/cm^3^) compared to the ($(10\bar{1}0)\text{-}\alpha$ surface (1.68 g/cm³). The flow flux under the $(10\bar{1}0)\text{-}\alpha$ surface is approximately 1.2 times higher than that under the $(10\bar{1}0)\text{-}\beta$ surface across various pressure gradients. We developed a theoretical model dividing the pore space into non-flowing, adsorbed, and bulk water regions, with critical thicknesses of 0.14 nm and 0.27 nm for the non-flowing region, and 0.15 nm for the adsorbed region in both surfaces. This model effectively predicts velocity distributions and volumetric flow rates with errors generally below 5%. Our findings provide insights into water transport mechanisms in shale inorganic nanopores and offer practical guidance for numerical simulation of shale gas production through dewatering operations.

## 1. Introduction

Shale oil and gas, as clean energy resources, have become a focal point in current exploration and development efforts to address energy challenges [1,2,3]. The shale formations that generate and store shale gas are characterized by ubiquitous nanoscale and microscale pores [4,5,6]. Large-scale hydraulic fracturing [7,8,9], involving high-pressure fluid injection into shale formations, serves as a crucial technique for stimulating tight rock matrices to release trapped gas and oil. However, the physical properties of fluids in confined nanoscale pores differ significantly from those under bulk conditions [10,11,12]. The confinement effects lead to various phenomena, such as adsorption [13,14], phase behavior changes [15,16], and slip effects [17,18], which play crucial roles in fluid transport. These nanoscale effects on fluids cannot be neglected. In nanopores, water–wall interactions can significantly influence water molecule distribution and confined fluid dynamic characteristics [19,20,21]. Therefore, to predict shale oil/natural gas development outcomes, it is imperative to understand the underlying mechanisms of water transport in shale nanoporous media [22,23].

Unlike conventional reservoirs, shale reservoirs contain both organic (e.g., kerogen) and inorganic (e.g., clay, quartz, and carbonates) materials [20,24], with both organic and inorganic pores featuring nanometer-scale pore sizes [25]. Based on geochemical analysis using X-ray diffraction (XRD) or fluorescence spectroscopy, quartz constitutes one of the primary components of shale rock [26,27,28], with Total Organic Carbon (TOC) typically comprising less than 20 wt% [29], while quartz can reach up to 65 wt% [30]. Consequently, studying the interactions between water and quartz surfaces becomes essential. The wettability of different α-quartz surfaces has been investigated through molecular simulation and contact angle measurements, demonstrating complete water-wet characteristics [31,32]. However, according to research by Kobayashi et al. [33], different quartz surfaces exhibit varying interactions with water, resulting in distinct water film structures. Regarding the influence of wettability on flow dynamics, Wu et al. [34] developed relational equations describing the relationship between wettability and slip length, establishing that stronger wettability corresponds to shorter slip lengths. Nevertheless, research by Ho et al. [35] revealed that slip phenomena can occur even on hydrophilic surfaces. These findings underscore the significance of studying water flow patterns on different α-quartz surfaces for shale oil and gas development.

The slip boundary condition has been widely applied to modify the Navier–Stokes (NS) equations to explain flow enhancement predicted by flow dynamics equations [36,37,38]. The critical thickness *δ* is commonly used to represent the thickness of the viscous region [34]. Zhan et al. [20] measured *δ* for various viscous minerals and found it approximately equals the thickness of the first adsorption layer. Fang et al. [39] proposed that *δ* should equal the thickness of the first water adsorption layer, which is approximately 0.29 nm in hydrophilic quartz nanopores. X-ray reflectivity experiments by Schlegel et al. [40] demonstrated that the real *α*-quartz $\left(10\bar{1}0\right)$ growth face exhibits two different terminations: the $(10\bar{1}0)\text{-}\alpha$ surface and the $(10\bar{1}0)\text{-}\beta$ surface, both possessing identical hydroxyl densities [33]. Chen et al. [21] modified graphene surfaces by adding hydroxyl groups and discovered that, at the same hydroxyl density, random versus uniform hydroxyl arrangements did not affect flow outcomes. However, Skelton et al. [41] indicated that the intrasurface hydrogen bonding (hydrogen bonding between OH of silanol groups at the surface) significantly influences surface hydration. The $(10\bar{1}0)\text{-}\beta$ surface exhibits higher intra-surface hydrogen bond density compared to the $(10\bar{1}0)\text{-}\alpha$ surface [41], resulting in different density distributions of water in the adsorption region [42]. Sun et al. [43] confirmed hydrogen bonding between hydroxyl groups influences the viscosity of surface-adsorbed water. Therefore, even on surfaces of identical minerals with equal surface hydroxyl densities, different phenomena may exist. Most flow studies have utilized only one type of quartz, leaving the characteristics of water flow and boundary conditions in different quartz nanopores not fully understood.

In this work, molecular dynamics (MD) simulations were employed to study water flow characteristics in two different terminations of *α*-quartz surfaces ($(10\bar{1}0)\text{-}\alpha$ and $(10\bar{1}0)\text{-}\beta$, which possess identical surface hydroxyl densities. Section 2 provides detailed descriptions of the quartz wall model construction, MD simulation parameters, and flow theory in nanoscale pores. Section 3 analyzes the distinct distribution characteristics of water molecules on $(10\bar{1}0)\text{-}\alpha$ and $(10\bar{1}0)\text{-}\beta$ surfaces, along with flow characteristics and flux, leading to the establishment of flow equations for different surfaces. Section 4 emphasizes key insights obtained from the simulations and provides a comprehensive evaluation of the results. This work provides more detailed mechanisms for water flow in inorganic nanopores of shale, offering different flow models for various shale formation scenarios with inorganic pores, thereby supporting numerical simulation and gas production through dewatering operations.

## 2. Models and Methods

In this section, we describe the molecular models and simulation methods for water flow in two different terminations of the *α*-quartz $\left(10\bar{1}0\right)$ surface ($(10\bar{1}0)\text{-}\alpha$ and $(10\bar{1}0)\text{-}\beta$ surfaces). All MD simulations were performed using the LAMMPS 2022 software package [44], with molecular configurations visualized using VMD 1.9.3 software [45].

### 2.1. Molecular Models

**Figure 1:**
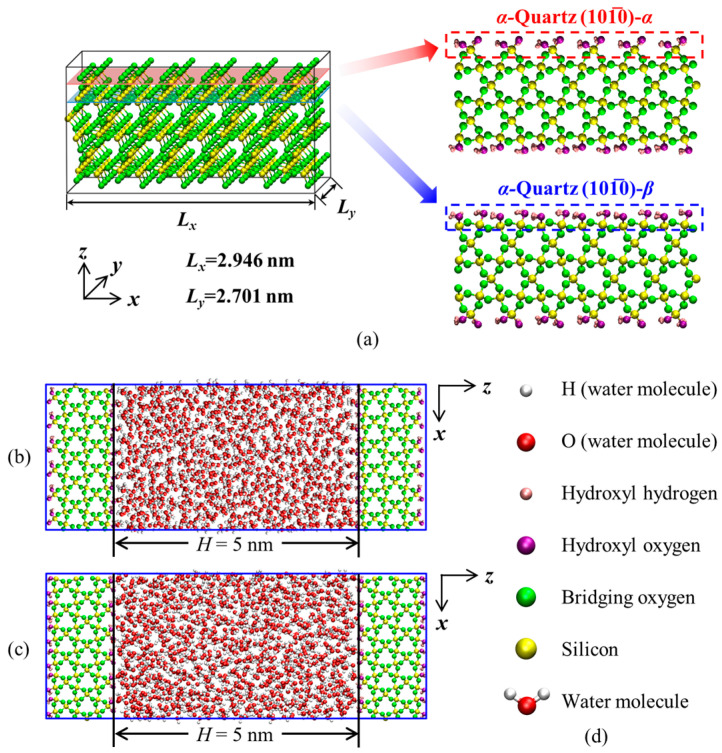
(**a**) The two different terminations of the *α*-quartz $\left(10\bar{1}0\right)$ surface. Snapshots of simulation systems with two different terminations as (**b**) $(10\bar{1}0)\text{-}\alpha$; (**c**) $(10\bar{1}0)\text{-}\beta$. *H* is the separation distance between the most inner hydroxyl oxygen atoms in the two quartz sheets. (**d**) Ball-and-stick model; white, red, pink, purple, green, and yellow spheres represent the hydroxyl SPC water model, H (water molecule), O (water molecule), hydroxyl H, hydroxyl O, bridging O, and Si, respectively.

Usually, the *α*-quartz $\left(10\bar{1}0\right)$ surfaces exhibit two different terminations [40]. The *α*-quartz unit was first cut along the $\left(10\bar{1}0\right)$ plane [41,42], followed by hydrogen addition to the cleaved oxygen atoms on the fractured quartz surface. The unit cell was then replicated and extended in the *x* and *y* directions to obtain the hydroxylated quartz surface [41]. Different surfaces were exposed based on where the surface was cleaved, as shown in Figure 1a. Figure 1 illustrates slit-shaped quartz nanopores composed of *α*-quartz $(10\bar{1}0)\text{-}\alpha$ and $(10\bar{1}0)\text{-}\beta$ surface structures as substrates. Figure 1b,c contain 1243 and 1259 water molecules, respectively. As sedimentary rocks were initially water-saturated, the substrate surfaces are fully hydroxylated to represent the strong hydrophilicity of quartz under geological conditions [46]. The pore width *H* is defined as the distance between the hydroxyl oxygen atoms on the inner sides of the two quartz surfaces. The dimensions of each substrate in the *x*, *y*, and *z* directions are 2.946 × 2.701 × 1.327 nm^3^, similar to the work of Wang et al. [46] and Xu et al. [47]. The hydroxyl (-OH) density on each surface is 7.54 sites/nm^2^, which aligns well with previous simulation work [48] and empirical calculations [49]. Furthermore, the quartz substrates remain rigid throughout all simulations, except for the hydroxyl hydrogens [50]. The ClayFF force field [51] is employed to describe the quartz nanopores, while water molecules are described using the SPC (Simple Point Charge) model [51].

### 2.2. Simulation Methods and Details

In this work, the temperature and pressure conditions are set at 323 K and 20 MPa, respectively. The target pressure is verified by comparing the bulk density of water at the pore center with the density under NPT ensemble conditions. This methodology is widely employed in MD simulations to determine the appropriate number of fluid molecules under confinement conditions [52,53,54]. Intermolecular interactions consist of the pairwise additive 12–6 Lennard-Jones (LJ) and Coulomb potentials. The LJ potentials are truncated at 1.2 nm with tail corrections. The Coulombic potential is used to model the long-range electrostatic interactions with the particle–particle particle–mesh (PPPM) method [46,55]. The interactions between different particles are described using the Lorentz–Berthelot mixing rules. Periodic boundary conditions are applied only in the *x* and *y* directions. In the *z*-direction, a large enough vacuum slab is added to minimize the periodic charge effects [39,56]. The time step is set at 1 fs.

First, 6 ns of equilibrium molecular dynamics (EMD) simulations are performed, with the initial 3 ns dedicated to system equilibration and the subsequent 3 ns for data sampling to analyze the static distribution characteristics of water molecules within the pore. Following the EMD simulations, external field non-equilibrium molecular dynamics (EF-NEMD) simulations are conducted by applying external driving forces to investigate water flow in quartz nanopores under pressure-driven conditions [57,58,59]. Referred to the previous research of Xu et al. [47], we employ a series of pressure gradients ranging from 1.5 to 9 MPa/nm to make the water form steady-state flow in the EF-NEMD simulations [60]. The total EF-NEMD simulation time is 9 ns, with the first 3 ns allocated for achieving steady-state flow conditions and the subsequent 6 ns for data collection.

## 3. Results and Discussions

### 3.1. Density Profile and Orientation Parameter

**Figure 2:**
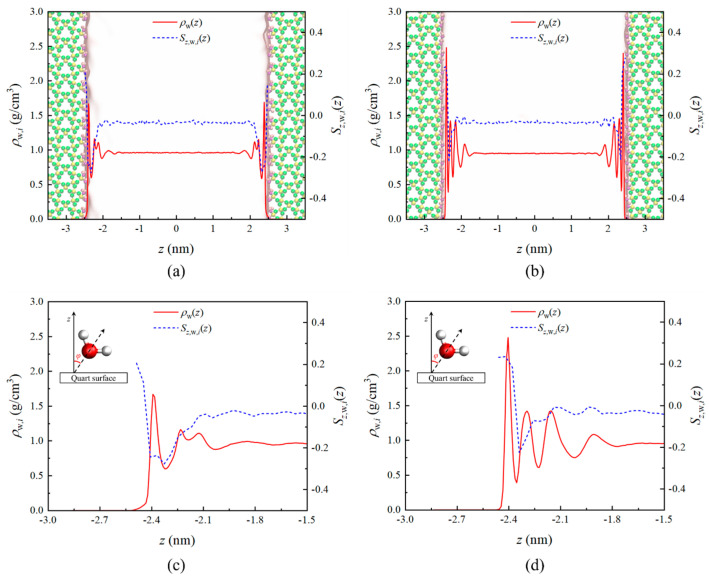
The density distribution *ρ*_w_ and orientation characteristics of water *S_z_*_,w,*i*_ at the two quartz surfaces: (**a**) $(10\bar{1}0)\text{-}\alpha$; (**b**) $(10\bar{1}0)\text{-}\beta$; (**c**) the region when −3.0 nm < *z* < 1.5 nm of $(10\bar{1}0)\text{-}\alpha$; (**d**) the region when 3.0 nm < *z* < 1.5 nm of $(10\bar{1}0)\text{-}\beta$.

Figure 2 illustrates the static density distribution of water molecules under confinement effects in quartz nanopores. In Figure 2a,b, due to the strong hydrophilicity of the quartz walls, water molecules exhibit four adsorption layers in both cases [39,61], with thicknesses of approximately 0.77 nm and 0.73 nm, respectively, which aligns with the critical thickness of the interfacial region determined by Wu et al. [34] and Xu et al. [47]. On the $(10\bar{1}0)\text{-}\alpha$ surface, the adsorption density of the first layer is 1.68 g/cm³, significantly lower than the 2.44 g/cm³ observed on the $(10\bar{1}0)\text{-}\beta$ surface, consistent with findings by Kobayashi et al. [33] and Skelton et al. [41]. Moreover, all four adsorption layers on the $(10\bar{1}0)\text{-}\alpha$ surface demonstrates lower densities compared to those on the $(10\bar{1}0)\text{-}\beta$ surface. The water density in the pore center remains relatively stable, with densities of 0.961 g/cm^3^ and 0.951 g/cm^3^ by MD simulation, respectively. These values show less than 5% relative error compared to the bulk water density of 0.996 g/cm^3^ under the same temperature and pressure conditions from the NIST database [62], thereby validating the accuracy of the force field employed.

Furthermore, the orientational parameter *S_z_*_,w,*i*_(*z*) of water molecules was calculated [63], which is given as

(1) $$S_{z,w,i}(z)=1.5\times \langle \cos^{2}\varphi \rangle -0.5,i=\alpha ,\beta ,$$

where *φ* is the angle of H_2_O between the *z*-axis and the connecting line of the O atom and the middle of two H atoms (as shown in Figure 2). *S_z_*_,w,*i*_(*z*) = 1 means that bisecting vectors of water molecule are fully perpendicular to the quartz surface, while *S_z_*_,w,*i*_(*z*) = −0.5 means that bisecting vectors of water molecule are fully parallel to the quartz surface, and *S_z_*_,w,*i*_(*z*) = 0 means that molecules have a disordered distribution.

**Figure 3:**
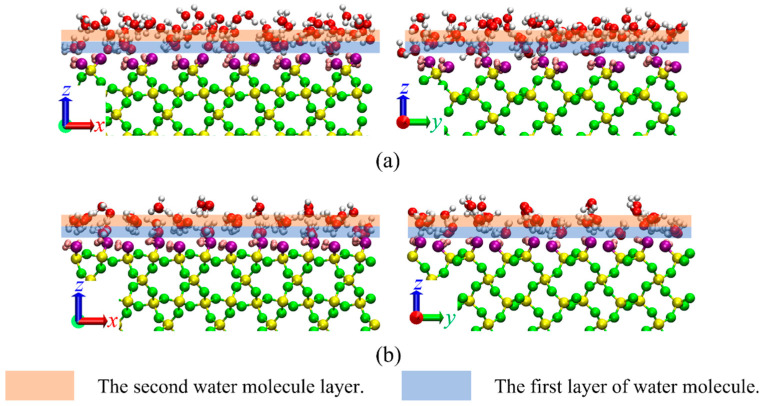
Snapshots of the water molecules near the two quartz surfaces: (**a**) $(10\bar{1}0)\text{-}\alpha$; (**b**) $(10\bar{1}0)\text{-}\beta$. The labels of *x*, *y* and *z* in the images mean the direction.

Figure 2 demonstrates the orientational parameter of water molecules, revealing strong orientational characteristics near the walls, indicating that water molecules form specific structures on the two quartz surfaces. As shown in Figure 2c, water molecules form only one layer arrangement structure under the $(10\bar{1}0)\text{-}\alpha$ surface; meanwhile, Figure 2d shows that water molecules form two layers of distinct arrangement structures under the $(10\bar{1}0)\text{-}\beta$ surface. To investigate this further, we obtained molecular simulation snapshots of the near-wall region, as illustrated in Figure 3. Water molecules near the $(10\bar{1}0)\text{-}\alpha$ surface show a relatively disordered distribution, while those near the $(10\bar{1}0)\text{-}\beta$ surface clearly exhibit a structure arranged in two layers, corresponding to the molecular orientation patterns.

### 3.2. Velocity Profile and Volume Flux

**Figure 4:**
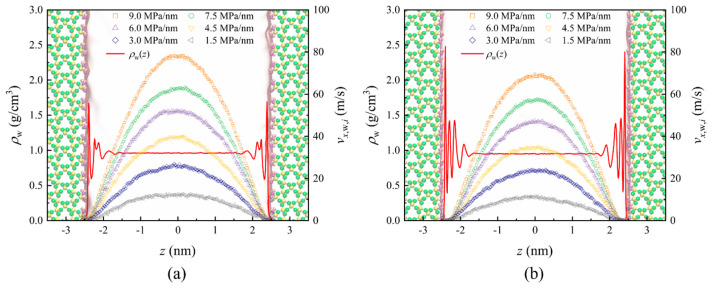
The density distribution *ρ*_w_ and the velocity profiles *v_x,w,i_* of water confined at the two quartz surfaces under various pressure gradients, (**a**) $(10\bar{1}0)\text{-}\alpha$ and (**b**) $(10\bar{1}0)\text{-}\beta$.

After establishing the static characteristics of water molecules in quartz nanopores, we conducted studies on water flow characteristics using EF-NEMD. Figure 4 shows the water velocity profiles and density distribution curves under different pressure gradients at the two quartz surfaces. The density distribution curves of the water phase under various pressure gradients essentially coincide with those under static conditions. This invariance of single-phase fluid density distribution concerning driving force has become a consensus in numerous EF-NEMD studies [17,46]. Under different pressure gradients, the water velocity profiles exhibit parabolic shapes, consistent with traditional Navier-Stokes (NS) equations descriptions. The flow velocity under the $(10\bar{1}0)\text{-}\alpha$ surface is observed to be slightly higher than that under the $(10\bar{1}0)\text{-}\beta$ surface. Two possible explanations for this phenomenon are first, the different flow boundaries under the two surfaces, and second, the varying viscosity of bulk water under the two surfaces.

Accordingly, we fitted the velocity distributions under different pressures for both surfaces using the equation *v_x,_*_w*,i*_(*z*) = *az*^2^ + *b*. In a single-phase pressure-driven Poiseuille flow, the viscosity *η*_w,bulk,*i*_, namely the shear viscosity, could be obtained according to the curvature of parabolic velocity profiles *v_x_*_,w*,i*_ [64],

(2) $$\eta_{w,bulk,i}=\frac{dp/dx}{\left(\frac{d^{2}v_{x,w,i}(z)}{dz^{2}}\right)},i=\alpha ,\beta ,$$

where *dp*/*dx* indicates the pressure gradient along the *x*-direction. Thus, the viscosity is obtained by $\eta_{w,bulk,i}=-\frac{dp/dx}{2a}$. Through fitting and calculations of the bulk water region velocities, the bulk water viscosity under the $(10\bar{1}0)\text{-}\alpha$ surface is 0.309 ± 0.016 mPa·s, while under the $(10\bar{1}0)\text{-}\beta$ surface it is 0.303 ± 0.014 mPa·s. These results closely align with the bulk viscosity value of 0.292 mPa·s calculated using the SPC water model at 19.1 MPa and 306.5 K [65], and show excellent agreement with the values of Zhan et al. (0.301 ± 0.026 mPa·s, 323 K, 20 MPa) [60]. This indicates that the velocity differences are solely attributable to differences in flow boundary conditions. Although Chen et al. [21] concluded that the distribution of hydroxyl groups does not influence the flow characteristics of water, the differences in atoms other than hydroxyl groups on the surface are the main reason for the differences in flow boundary conditions, as shown in Figure 1, Figure 2 and Figure 3.

**Figure 5:**
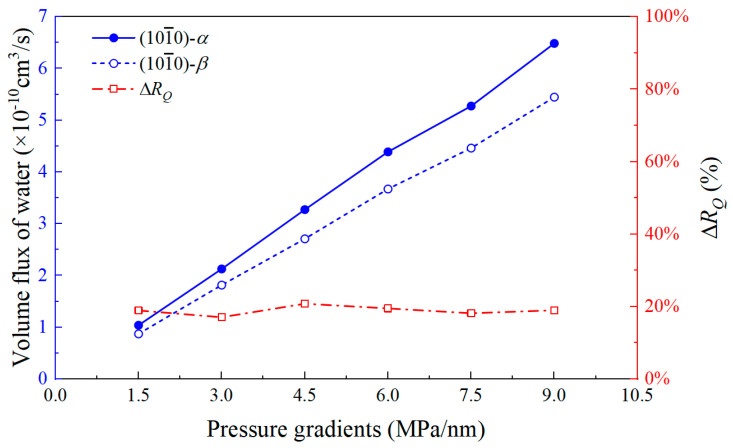
Volume flux of water confined in two quartz nanopores under various pressure gradients and the volume flow deviation (Δ*R_Q_*).

To quantify the effects of velocity differences, we calculated the respective flow flux [56],

(3) $$J_{w,i}^{\mathrm{MD}}=\frac{\int_{-\frac{H}{2}}^{\frac{H}{2}}\rho_{w,i}(z)v_{x,w,i}(z)dz}{\rho_{w,\mathrm{bulk}}},i=\alpha ,\beta ,$$

where *ρ*_w,bulk_ is the bulk density of water at the given temperature and pressure conditions, g/cm^3^. Using the flow flux under the $(10\bar{1}0)\text{-}\beta$ wall as the reference, the difference in flow flux between the two quartz surfaces is quantified ($\Delta R_{Q}=\frac{J_{w,\alpha }^{\mathrm{MD}}-J_{w,\beta }^{\mathrm{MD}}}{J_{w,\beta }^{\mathrm{MD}}}\times 100\%$), as shown in Figure 5. It can be observed that the flow flux under both wall surfaces exhibits a good linear relationship with the pressure gradient. The flow flux under the $(10\bar{1}0)\text{-}\alpha$ wall is consistently higher than that under the $(10\bar{1}0)\text{-}\beta$ wall for any pressure gradient. A comparison of the flow flux differences shows that the flow flux under the $(10\bar{1}0)\text{-}\alpha$ wall is approximately 1.2 times that under the $(10\bar{1}0)\text{-}\beta$ wall. Therefore, the study of flow boundaries is crucial, and the next section will present the flow boundaries for the two wall surfaces.

### 3.3. Water Flow Theoretical Model

**Figure 6:**
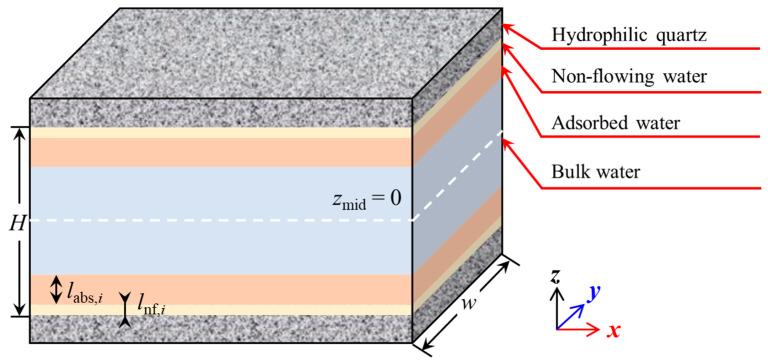
Schematic representation of water flow in quartz nanopores. *H* is the pore width in the *z* direction, *z*_mid_ is the *z*-coordinate of the middle point in pore space, *l*_abs,*i*_ is the critical thickness of the adsorbed water region, *l*_nf*,i*_ is the critical thickness of the non-flowing water region, and *w* is the channel width in the *y* direction.

The mathematical model based on the continuous medium hypothesis has been widely applied in the petroleum industry. Although the fluid density within nanoscale confined spaces exhibits heterogeneity due to the influence of pore surfaces, with a continuous variation in density distribution, many researchers simplify the calculation by considering the effects of solid–liquid interactions on the fluid density distribution. They divide the fluid-filled space inside the pore into two regions: the near-wall region and the bulk water region [22,34,37,66,67]. Based on the analysis in the previous section, the water fluid is divided into three regions: the non-flowing water region, the adsorbed water region, and the bulk water region, as shown in Figure 6. Each region exhibits different fluid physical properties, such as density, viscosity, and others.

The critical thickness (*l*_nf*,i*_) of the non-flowing water region is determined based on the velocity profile and adsorption layer thickness shown in Figure 4, measuring 0.14 nm beneath the $(10\bar{1}0)\text{-}\alpha$ surface and 0.27 nm beneath the $(10\bar{1}0)\text{-}\beta$ surface. Regarding the critical thickness (*l*_abs,*i*_) of the adsorbed water region, some researchers consider it to be the thickness of all fluid adsorption layers, taking *l*_abs,*i*_ as 0.7 nm or 0.8 nm [68,69], while others argue it should be the thickness of the first adsorption layer, approximately 0.3 nm [60]. To determine a reasonable value for this thickness, this study establishes a mathematical model for single-phase water flow based on continuous medium assumptions. By combining this with velocity distribution curves obtained from molecular simulations, various current perspectives are analyzed and compared to determine the precise thickness of the adsorbed water region during single-phase water flow in quartz nanopores.

For the non-flowing water region, the velocity is assumed to be zero, indicating no flow occurs.

For the adsorbed water region, according to the theory of Wu et al. [34], the viscosity (*η*_w,abs_), density (*ρ*_w,abs_), and slip length (*l_s_*) are all dependent on wettability, are given as

(4) $$\frac{\eta_{w,\mathrm{abs}}}{\eta_{w,\mathrm{bulk}}}=-0.018\theta +3.25,$$

(5) $$\rho_{w,\mathrm{abs}}=\rho_{w,\mathrm{bulk}}+(1.6-0.0095\theta ),$$

(6) $$l_{s}=\frac{0.41}{{\left(1+\cos\theta \right)}^{2}},$$

where *θ* is the contact angle of water on the quartz surface. According to the research by Kobaya et al. [33], the systems of the $(10\bar{1}0)\text{-}\alpha$ surface and $(10\bar{1}0)\text{-}\beta$ surface are completely water-wet, that is, the contact angles are zero. Then, the velocity of water in the adsorbed water region *v_x_*_,w,*i*_(*z*) is given as

(7) $$v_{x,w,i}\left(z\right)=\frac{1}{2\eta_{w,\mathrm{abs}}}\frac{dp}{dx}\left({\left(\frac{H}{2}-l_{\mathrm{nf},i}\right)}^{2}-z^{2}\right)+C_{1,i},\frac{H}{2}-l_{\mathrm{nf},i}-l_{\mathrm{abs},i}\leq \left|z\right|\leq \frac{H}{2}-l_{\mathrm{nf},i}$$

For bulk water region, the velocity of water *v_x_*_,w,*i*_(*z*) is given as

(8) $$v_{x,w,i}\left(z\right)=\frac{1}{2\eta_{w,\mathrm{bulk}}}\frac{dp}{dx}\left({\left(\frac{H}{2}-l_{\mathrm{nf},i}\right)}^{2}-z^{2}\right)+C_{2,i},0\leq \left|z\right|\leq \frac{H}{2}-l_{\mathrm{nf},i}-l_{\mathrm{abs},i}$$

Then, considering the slip boundary at the liquid–wall interface and assuming that the water velocity distribution at the absorbed water and bulk water interface is continuous, the constants *C*_1,*i*_ and *C*_2,*i*_ in Equations (9) and (10) are given as

(9) $$C_{1,i}=\frac{l_{s}(\frac{H}{2}-l_{\mathrm{nf},i})}{\eta_{w,\mathrm{abs}}}\frac{dp}{dx}$$

(10) $$C_{2,i}=\left(\frac{1}{2\eta_{w,\mathrm{abs}}}-\frac{1}{2\eta_{w,\mathrm{bulk}}}\right)\frac{dp}{dx}l_{\mathrm{abs},i}\left(l_{\mathrm{abs},i}+2l_{\mathrm{nf},i}-H\right)+C_{1,i}$$

where *i* = *α*, *β* represent the $(10\bar{1}0)\text{-}\alpha$ surface and the $(10\bar{1}0)\text{-}\beta$ surface, respectively.

**Figure 7:**
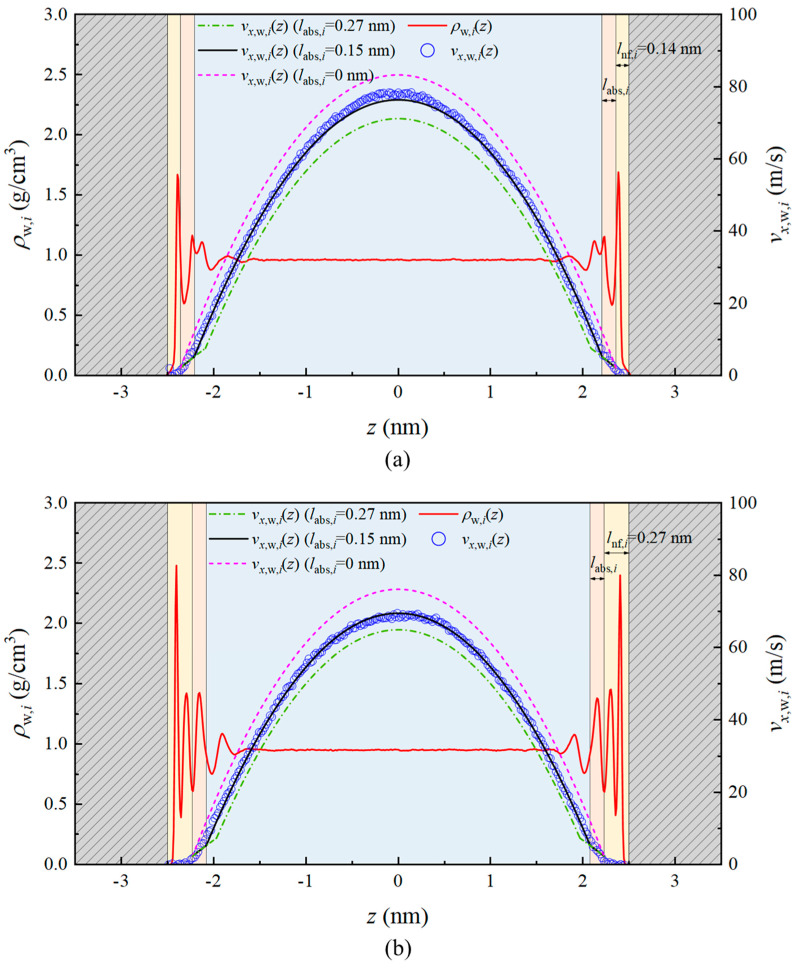
The density distribution *ρ*_w_ and the velocity profiles in *x*-direction *v_x_*_,w,*i*_(*z*) of water in two quartz nanopores under 9 MPa/nm by using various *l*_abs,_ values: (**a**) $(10\bar{1}0)\text{-}\alpha$; (**b**) $(10\bar{1}0)\text{-}\beta$.

We set the values of *l*_abs,*i*_ to 0 nm, 0.15 nm, and 0.27 nm, with the corresponding velocity distributions shown in Figure 7. Under the $(10\bar{1}0)\text{-}\alpha$ surface, the *l*_nf*,α*_ value of 0.14 nm encompasses only the first adsorption layer, while under the $(10\bar{1}0)\text{-}\beta$ surface, the *l*_nf*,β*_ value of 0.26 nm spans both the first and second adsorption layers. Notably, the hydroxyl density is identical for both surfaces, with the only difference being the arrangement of the hydroxyl groups. According to the theory of Ho et al. [35], if adsorption sites on a hydrophilic surface are sufficiently close to allow water molecules to easily migrate from one site to another, such hydrophilic surfaces can exhibit liquid slip. Thus, the hydroxyl arrangement pattern can account for these observed differences. Comparing with the water molecule velocity profiles obtained from MD simulations, we found that setting *l*_abs_ to 0.15 nm provides the best fit (with relative errors of 1.18% for $(10\bar{1}0)\text{-}\alpha$ and 0.11% for $(10\bar{1}0)\text{-}\beta$). This thickness approximates that of the next adsorption layer beyond the non-flowing water region. Using the 0 nm model overestimates the average velocity (with relative errors of 12.17% for $(10\bar{1}0)\text{-}\alpha$ and 14.36% for $(10\bar{1}0)\text{-}\beta$), while the 0.27 nm model underestimates it (with relative errors of −6.06% for $(10\bar{1}0)\text{-}\alpha$ and −4.30% for $(10\bar{1}0)\text{-}\beta$).

**Figure 8:**
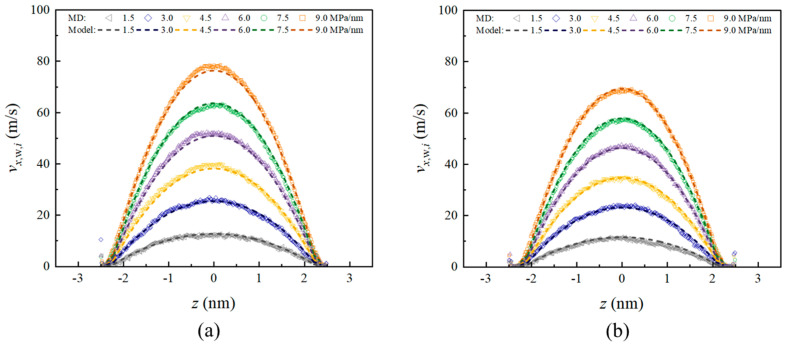
Liquid velocity profiles from the model and MD simulations at various pressure gradients (MPa/nm) for (**a**) $(10\bar{1}0)\text{-}\alpha$; (**b**) $(10\bar{1}0)\text{-}\beta$.

Accordingly, we selected 0.15 nm as *l*_abs,*i*_ for both cases, while using 0.14 nm and 0.27 nm, respectively, for *l*_nf*,i*_ to calculate velocity distributions under different pressure gradients, as shown in Figure 8. The results demonstrate that this model can effectively predict velocity distributions under different wall surfaces.

Furthermore, we calculated the model flow flux. In the model, the water mass fluxes in the adsorbed water region, *Q*_abs,*i*_, in the bulk water region *Q*_bulk,*i*_ are given as

(11) $$Q_{\mathrm{abs},i}=2\rho_{w,\mathrm{abs}}\int_{\frac{H}{2}-l_{\mathrm{nf},i}-l_{\mathrm{abs},i}}^{\frac{H}{2}-l_{\mathrm{nf},i}}v_{x,w,i}\left(z\right)wdz=\frac{\rho_{w,\mathrm{abs}}}{\eta_{w,\mathrm{abs}}}\frac{dp}{dx}w\left({\left(\frac{H}{2}-l_{\mathrm{nf},i}\right)}^{2}-\frac{2}{3}l_{\mathrm{abs},i}^{2}+c_{1,i}\right)l_{\mathrm{abs},i}$$

(12) $$Q_{\mathrm{bulk},i}=2\rho_{w,\mathrm{bulk}}\int_{0}^{\frac{H}{2}-l_{\mathrm{nf},i}-l_{\mathrm{abs},i}}v_{x,w,i}\left(z\right)wdz=\frac{\rho_{w,\mathrm{bulk}}}{3\eta_{w,\mathrm{bulk}}}\frac{dp}{dx}w(\frac{H}{2}-l_{\mathrm{nf},i}-l_{\mathrm{abs},i})\left({\left(\frac{H}{2}-l_{\mathrm{nf},i}\right)}^{2}+4(\frac{H}{2}-l_{\mathrm{nf},i})l_{\mathrm{abs},i}-2l_{\mathrm{abs},i}^{2}+3c_{2,i}\right)$$

where *c*_1,*i*_, *c*_2,*i*_ are given as

(13) $$c_{1,i}=\frac{C_{1,i}}{\left(\frac{1}{2\eta_{w,\mathrm{abs}}}\frac{dp}{dx}\right)}=2l_{s}(\frac{H}{2}-l_{\mathrm{nf},i})$$

(14) $$c_{2,i}=\frac{C_{2,i}}{\left(\frac{1}{2\eta_{w,\mathrm{bulk}}}\frac{dp}{dx}\right)}=\left(\frac{\eta_{w,\mathrm{bulk}}-\eta_{w,\mathrm{abs}}}{\eta_{w,\mathrm{abs}}}\right)l_{\mathrm{abs},i}\left(l_{\mathrm{abs},i}+2l_{\mathrm{nf},i}-H\right)+c_{1,i}\frac{\eta_{w,\mathrm{bulk}}}{\eta_{w,\mathrm{abs}}}$$

where *w* = 2.701 nm is the width of the pore space in the *y*-direction. The total volumetric flux of water using the model, *J*_w,*i*_ is given as

(15) $$J_{w,i}=\left(Q_{\mathrm{abs},i}+Q_{\mathrm{bulk},i}\right)/\rho_{w,\mathrm{bulk}}$$

To verify the accuracy of the model, we calculated the relative error (Δ*ε_i_*) between the model volumetric flow flux and MD simulation volumetric flow flux, which is given as

(16) $$\Delta \varepsilon_{i}=\frac{\left|J_{w,i}-J_{w,i}^{\mathrm{MD}}\right|}{J_{w,i}^{\mathrm{MD}}}\times 100\%,i=\alpha ,\beta ,$$

**Figure 9:**
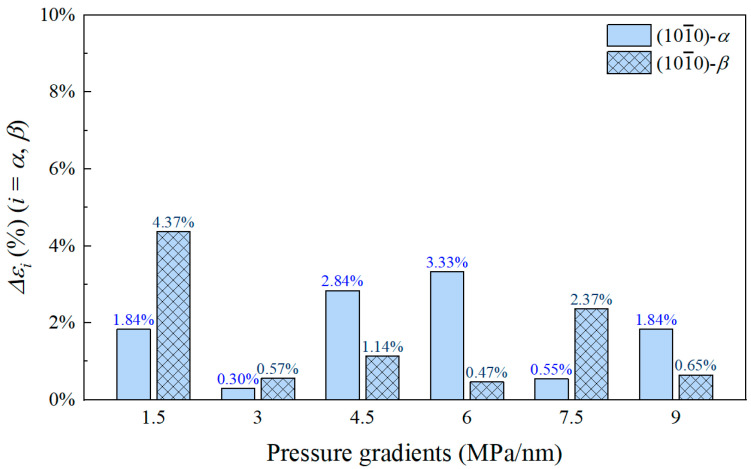
The relative errors Δ*ε_i_* of water volume fluxes in two quartz nanopores calculated by the model.

The calculated errors are shown in Figure 9. As can be seen, our model accurately calculates the volumetric fluxes of water in the two quartz nanopores, with relative errors within 5%. Based on the comparison of the flow data, our established model can effectively predict both velocity distributions and volumetric flow rates in pores with different quartz surfaces.

## 4. Conclusions

In this study, we investigated water flow characteristics in different *α*-quartz $\left(10\bar{1}0\right)$ surface nanopores using molecular dynamics simulations. We examined two distinct surface terminations ($(10\bar{1}0)\text{-}\alpha$ and $(10\bar{1}0)\text{-}\beta$), which possess identical hydroxyl densities but different arrangements. Through equilibrium and non-equilibrium molecular dynamics simulations, we analyzed the density distribution, molecular orientation, velocity profiles, and volumetric flux of confined water, and based on MD simulation results, a hydrodynamic flow model to describe water in nanopores was developed. The detailed conclusions are listed as following.

1. Despite having identical hydroxyl densities, the $(10\bar{1}0)\text{-}\alpha$ and $(10\bar{1}0)\text{-}\beta$ surfaces exhibit distinct water molecule distribution patterns. The $(10\bar{1}0)\text{-}\beta$ surface shows stronger water molecule structuring with a structure arranged in two layers and higher first-layer adsorption density (2.44 g/cm³) compared to the more disordered distribution and lower first-layer density (1.68 g/cm³) on the $(10\bar{1}0)\text{-}\alpha$ surface;

2. The flow characteristics differ significantly between the two surfaces. Under identical pressure gradients, the flow flux through $(10\bar{1}0)\text{-}\alpha$ surface nanopores is consistently about 1.2 times higher than through $(10\bar{1}0)\text{-}\beta$ surface nanopores. This difference is primarily attributed to varying flow boundary conditions rather than bulk water viscosity, which remains similar for both surfaces (0.309 ± 0.016 mPa·s and 0.303 ± 0.014 mPa·s, respectively);

3. We established a theoretical model that divides the pore space into three regions: non-flowing water, adsorbed water, and bulk water. The critical thickness of the non-flowing region is 0.14 nm for the $(10\bar{1}0)\text{-}\alpha$ surface and 0.27 nm for the $(10\bar{1}0)\text{-}\beta$ surface, while the adsorbed region thickness is 0.15 nm for both surfaces. This model effectively predicts both velocity distributions and volumetric flow rates with errors generally below 5%;

4. The findings demonstrate that surface hydroxyl arrangement patterns significantly influence water flow characteristics in quartz nanopores, even when hydroxyl densities are identical. This has important implications for understanding and predicting fluid transport in shale formations with varying mineral surface properties.

These results provide valuable insights into water transport mechanisms in shale inorganic nanopores and offer practical guidance for numerical simulation of shale gas production through dewatering operations. The developed theoretical model can be applied to improve the accuracy of large-scale reservoir simulations.

## Data Availability

Data are contained within the article.
